# PIBF+ extracellular vesicles from mouse embryos affect IL-10 production by CD8+ cells

**DOI:** 10.1038/s41598-018-23112-z

**Published:** 2018-03-16

**Authors:** Eva Pallinger, Zoltan Bognar, Agnes Bogdan, Timea Csabai, Hajnalka Abraham, Julia Szekeres-Bartho

**Affiliations:** 1 Department of Genetics, Cell- and Immunobiology, Semmelweis University, Budapest, Hungary; 2 Department of Medical Biology, Medical School, Pecs University, Pecs, Hungary; 3 MTA - PTE Human Reproduction Research Group, Hungarian Academy of Science at Pecs University, Pecs, Hungary; 4 János Szentágothai Research Centre, Pecs University, Pecs, Hungary; 5 Endocrine Studies, Centre of Excellence, Pecs University, Pecs, Hungary

## Abstract

Earlier evidence suggests, that the embryo signals to the maternal immune system. Extracellular vesicles (EVs) are produced by all types of cells, and because they transport different kinds of molecules from one cell to the other, they can be considered as means of intercellular communication. The aim of this work was to test, whether the embryo is able to produce sufficient amounts of EVs to alter the function of peripheral lymphocytes. Embryo-derived EVs were identified by their Annexin V biding capacity, and sensitivity to Triton X dependent lysis, using flow cytometry. Transmission electron microscopy was used to detect EVs at the implantation site. Progesterone-induced blocking factor (PIBF) expression in embryo-derived EVs was demonstrated with immuno-electron microscopy. The % of IL-10 + murine lymphocytes was determined by flow cytometry. EVs were present in embryo culture media, but not in empty media. Mouse embryo-derived EVs adhere to the surface of both CD4+ and CD8+ murine peripheral T lymphocytes, partly, via phosphatidylserine binding. The number of IL-10+ murine peripheral CD8+ cells increases in the presence of embryo-derived EVS, and this effect is counteracted by pre-treatment of EVs with an anti-PIBF antibody, suggesting that the embryo communicates with the maternal immune system via EVs.

## Introduction

Pregnancy has a profound influence on the functioning of the maternal immune system. Owing to the concerted action of NK cells, regulatory T cells and altered cytokine balance, the developing embryo enjoys a favourable immunological environment throughout gestation. Though later stages of pregnancy have been relatively well characterized in this respect, little is known about the embryo-maternal interactions in the peri-implantation period.

Earlier data suggest, that such an early communication might exist. Daya and Clark demonstrated immunosuppressive factors in embryo culture medium^1^ and Kelemen *et al*.^2^ reported increased IL-10 mRNA expression in peripheral lymphocytes incubated with the culture media of fertilized eggs, but not in those, incubated with follicular fluid.

Thus, there is evidence, that the embryo releases signals, to alter the maternal immune functions, from the earliest stages of pregnancy, however, the mechanism of signal transport has not been thoroughly investigated.

In recent years extracellular vesicles (EVs) have received much attention. These membrane-coated structures may express phosphatidylserine (PS) in their membrane^3^, which reacts with Annexin V.

EVs are produced by all types of cells, and because they transport different kinds of molecules from one cell to the other, they can be considered as means of intercellular communication, and as such, might be considered as candidates for conveying the signal from the embryo to the mother. Earlier, we showed that *in vitro* cultured human embryos produce detectable numbers of EVs^4^, therefore, it seemed plausible, that these structures might be involved in the communication between the embryo and the endometrium during implantation.

EVs originating from various cell types and carrying different molecules can both activate and suppress the function of the immune system, by presenting antigens^5,6^, MHC molecules^7–10^ or cytokines^11–16^.

The Progesterone-induced Blocking factor (PIBF) was originally described as a 34 kDa protein produced by peripheral pregnancy lymphocytes. Later it became obvious, that PIBF is expressed by many other cell types and plays a role in the feto-maternal communication, partly, by mediating the immunological actions of progesterone^17^.

The aim of this work was to test, whether the embryo-derived EVs might carry PIBF, and whether PIBF+ embryo-derived EVs might alter the function of peripheral lymphocytes, this way contributing to the communication between the embryo and the mother in the early stage of pregnancy.

## Materials and Methods

### Embryo culture

Eight to 12 weeks old CD1 female mice (Charles River, Germany) were injected with 5 IU of FSH (Merional, IBSA Pharma, Switzerland). Forty eight hours later the mice were treated with 5 IU LH (Chloragon, Ferring, Hungary), and directly placed to CD1 males. Twenty four hours after sighting the vaginal plug, two cell stage embryos were flushed from the fallopian tubes, and cultured individually in 50 μl droplets in KSOM medium (Millipore, England), supplemented with 0.4% of BSA, under mineral oil at 37 C°, 5% CO2, for 72 h, until they reached the blastocyst stage. Culture media were replaced every 24 hours. After 24 h culture, mouse embryos are at the 6–8 cell stage, during a further 24 h of culture they develop into morulae, and an additional 24 h culture period is needed for the embryos to reach the blastocyst stage. At this point the culture media of individual blastocysts were collected, and stored at −80 ^o^C, until used. Media from embryos collected at earlier stages of development were not used in this study.

All methods were carried out in accordance with relevant guidelines and regulations. All experimental protocols were approved by the Animal Health Committee of Baranya County.

### Flow cytometry

Measurements were carried out using a BD FACSCalibur (BD Biosciences, San Jose, USA) flow cytometer, and data were analyzed with CellQuestPro software. The instrument settings and gates were defined by Megamix-Plus SSC beads (Biocytex, France) and were optimized with 1 µm Silica Beads Fluo-Green Green (Kisker Biotech GmbH & Co; Steinfurt, Germany). The single-platform flow cytometric determination of the absolute number of EVs was performed by adding internal counting standard beads (Sysmex Partec GmbH; Germany) to embryo culture medium samples. The absolute number of EVs was calculated using the following formula:

$$\begin{array}{rcl}{\rm{Absolte}}\,{\rm{EV}}\,{\rm{Count}}\,(\text{ENs}/{\rm{\mu }}{\rm{L}}) & = & ({\rm{Number}}\,{\rm{of}}\,{\rm{EV}}\,\text{event}/\text{Number}\,{\rm{of}}\,{\rm{bead}}\,{\rm{event}})\\  &  & \,\times {\rm{Concentration}}\,{\rm{of}}\,{\rm{bead}}\,(\text{bead}/{\rm{L}})\end{array}$$

### EVs were identified by Annexin V binding to PS expressed by the vesicle membranes

For Annexin V staining 2 μl of embryo culture medium was diluted with 250 μl of annexin binding buffer (BD Biosciences, San Jose, USA) and incubated for 10 minutes at room temperature with 1 μl of phycoerythrin conjugated AnnexinV (BD Biosciences, San Jose, USA). Fifty μl Count Check Beads (Sysmex Partec GmbH) was also added for determination of the number of EVs. To confirm the presence of EVs, we applied Triton-X differential detergent lysis using a final concentration of 0.1% as described by György *et al*.^18^. Because the detergent lyses the membrane of the vesicles, events that disappeared in the presence of 0.1% Triton-X 100 were considered as EVs^18,19^. Empty medium (culture medium that did not contain embryos, but was otherwise treated under the same conditions as embryo culture medium) was used as negative control.

### Electron microscopy

The presence of EVs at the feto-maternal interface in day 5 murine pregnancy was confirmed using transmission electron microscopy. Approximately 1 mm^3^ uterine tissues from day 5 murine pregnancy were washed in PBS, fixed with 2.5% glutaraldehyde diluted in phosphate buffer (0.1 M, pH 7.4) overnight at 4 °C. After washing with PBS, the blocks were post-fixed in 1% osmium tetroxide in 0.1 M PBS for 1 hour at 4 °C and dehydrated with increasing concentration of ethanol. After complete dehydration, blocks were transferred into propylene oxide 2 times for 4 minutes. Following this, blocks were immersed in the mixture of propylene oxide and Durcupan resin (Sigma, St Louis, USA) for 30 minutes. Then, blocks were placed into Durcupan-containing aluminum-foil boats overnight, and embedded into gelatin capsule filled with Durcupan resin. After polymerization and hardening of the resin at 56 °C for 72 hours, 700 nm semi-thin sections were cut with Leica Ultracut ultramicrotom, mounted on glass slides, stained with toluidine- blue and examined with Olympus BX50 light microscope. Following this, 65 nm serial ultrathin sections were cut with the ultramicrotome, and mounted on mesh and single-slot copper grids. The ultrathin sections were then contrasted by uranyl acetate and lead citrate, and were examined in JEOL 1200EX-II electron microscope.

### Immuno-electron microscopy

*In vitro* cultured morula stage mouse embryos were stained in droplet. The embryos were fixed in 4% formaldehyde buffered in PB for 20 minutes at room temperature. Following fixation, blocking of endogenous peroxidase was achieved by immersing the embryos in 1% hydrogen peroxide for 15 minutes, non-specific binding sites were blocked with 3% of bovine serum albumin for 40 minutes. Embryos were then reacted with 1:50 diluted rabbit anti-PIBF primary antibody^20^ for 2 hours at room temperature. Polyclonal anti-PIBF antibody was generated in our laboratory by immunizing rabbits with the 48-kDa N-terminal part of the human recombinant PIBF. The IgG from immune sera was affinity purified on protein-A or protein-G columns (AP Hungary Ltd, Budapest, Hungary). The antibody titres were determined by ELISA using the recombinant PIBF protein as the antigen. An aliquot of the antibody was absorbed with the 48-kDa N terminal part of the recombinant PIBF to be used for testing the specificity of binding on Western blots^20^. Binding of the primary antibody was detected with horseradish peroxidase conjugated anti-rabbit secondary antibody (1:100) (Dako, Denmark) at room temperature for 1 hour. Peroxidase reaction was visualized using diaminobenzidine as chromogene. Following the immunoreaction, embryos were mixed in 3% agar solution ((Sigma, St Louis, USA). After agar was hardened, small blocks of approximately 1 mm^3^ were cut and fixed with 2.5% glutaraldehyde diluted in PB overnight at 4 °C. Post-fixation with 1% osmium tetroxide, embedding and cutting of the blocks were performed as described above.

### Binding of EVs to mouse spleen cells

Spleen cells of 16 weeks old female CD1 mice were prepared by mechanical separation and filtered through a 30 μm Filcon filters (BD Biosciences, San Jose, USA). Multicolor staining method was used for the detection of CD4+ Th and CD8+ Tc cells. All antibodies (APC conjugated anti-mouse CD4 cat No: 553051 Clone RM4–5 (RUO) 553051or PeCy7 conjugated anti-mouse CD8 cat. No: 552877 Clone 53–6.7 (RUO) 552877) were purchased from BD Biosciences.

For visualization the phosphatidylserine receptor (PSR) on CD4+ and CD8+ cells, the cells were reacted with anti-phosphatidylserine receptor antibody (Sigma; Cat no: P1495) for 20 min at room temperature, and with FITC-labelled secondary antibody (anti-rabbit IgG; Sigma; dilution 1: 50 with antibody buffer) for 30 min at room temperature. FITC-labelled secondary antibody was used as negative control to distinguish non-specific background signal from specific antibody signal.

Extracellular vesicles (EV fraction = 12.5 K pellet) were sedimented from five pooled mouse embryo culture media at 12,500 g for 20 minutes at 16◦C (Z216 M K Microlite centrifuge, 200.88 rotor, Hermle Labortechnik) and subsequently washed at 12,500 g for 15 minutes at 16◦C. Separated EVs were labelled with freshly prepared PKH-26 fluorescent dye at a final concentration of 5 μM of PKH according to the instructions of the manufacturer (Sigma, St Louis, USA).

PKH–labelled EVs were incubated in a 96 well plate with 5 × 10^4^ anti-CD4 and anti-CD8 labelled splenocytes in the presence of 4% BSA for 30 minutes at 4 °C (BSA was used to inhibit binding of excess PKH to the lymphocytes). Diluted PKH dye solution without EVs (mock-stained control) was used to assess non-specific (EV-independent) staining (supplementary Fig. 1). At the end of the incubation period, cells were washed with PBS and analysed by flow cytometry. Experiments and measurements were repeated six times in two parallels. Measurements were carried out using a FACSCalibur flow cytometer (Becton Dickinson, CA, USA) on the day of the staining, collecting 2.5 × 10^4^ − 1 × 10^5^ cells/tube. CellQuest-Pro software (Becton Dickinson, CA, USA) was used for analysis.

### Inhibition of EV binding to lymphocytes

To investigate the mechanism of EV binding to the lymphocytes, we measured EV binding after reacting the exofacial phosphatidylserine (PS) of embryo-derived EVs by Annexin V, or masking the surface phosphatidylserine receptor (PSR) molecules of splenocytes with anti-PSR polyclonal antibody.

CD4 and CD8 labelled splenocytes were incubated with 1 μg/ml of polyclonal anti-phosphatidylserine receptor antibody (Sigma; Cat no: P1495) for 20 min, at room temperature.

PKH labelled embryo-derived EVs were incubated with 1 μl of Annexin V (Sony Biotechology Inc) in Annexin binding buffer for 5 minutes at room temperature.

Embryo-derived (untreated, or Annexin V treated) EVs were incubated with untreated or anti-PSR-treated lymphocytes in PBS, 4% BSA for 30 minutes at 4 ^o^C.

PKH fluorescence signal of CD4+ and CD8+ lymphocytes was detected.

### Intracellular IL-10 staining of splenocytes

Five hundred thousand splenocytes were stimulated with embryo-derived EVs for 4 hours in the presence of the protein transport inhibitor brefeldin A (Sigma, St Louis, USA). Stimulated cells were washed and incubated in 50 µl of staining buffer (1% BSA/PBS) with a pre-titrated optimal concentration of allophycocyanin (APC)-conjugated rat anti-mouse CD4 (APC Rat Anti-Mouse CD4 Clone RM4-5 (RUO) 553051), and FITC-conjugated rat anti-mouse CD8 (PE-Cy™7 Rat Anti-Mouse CD8a Clone 53-6.7 (RUO) 552877) monoclonal antibodies (15–30 min, at room temperature) for identifying CD4+ and CD8+ lymphocytes.

Anti-CD4 and anti-CD8 labelled cells were fixed (4% paraformaldehyde solution for 10 minutes, at room temperature), permeabilized with 0.1% saponin and incubated in 50 µl of staining buffer (PBS containing 1% BSA) with a pre-titrated optimal concentration of phycoerythrin-conjugated anti-mouse IL-10 monoclonal antibody (PE Rat Anti-Mouse IL-10 Clone JES5-16E3 (RUO) 561060) for 15–30 min, at room temperature. The cells were washed twice in 2 ml of PBS, centrifuged for 5 min at 300 g, and fixed with 300 μl of 2% paraformaldehyde (Sigma, St Louis, USA). Stained cells were stored at 4 °C in dark before analysis. All fluorochrome-conjugated monoclonal antibodies were purchased from BD Biosciences. Tests were carried out by measuring, 5 × 10^4^ cells/tube, on the day of the staining. All the FACS data were analysed with CellQuestPro software (Becton Dickinson, CA, USA).

### Statistics

The two-tailed Student’s t-test was used for statistical evaluation of the data. Differences were considered significant if the *P* value was equal to or less than 0.05.

## Results

### Mouse embryos produce extracellular vesicles. Mouse embryo-derived extracellular vesicles contain PIBF

**Figure 1:**
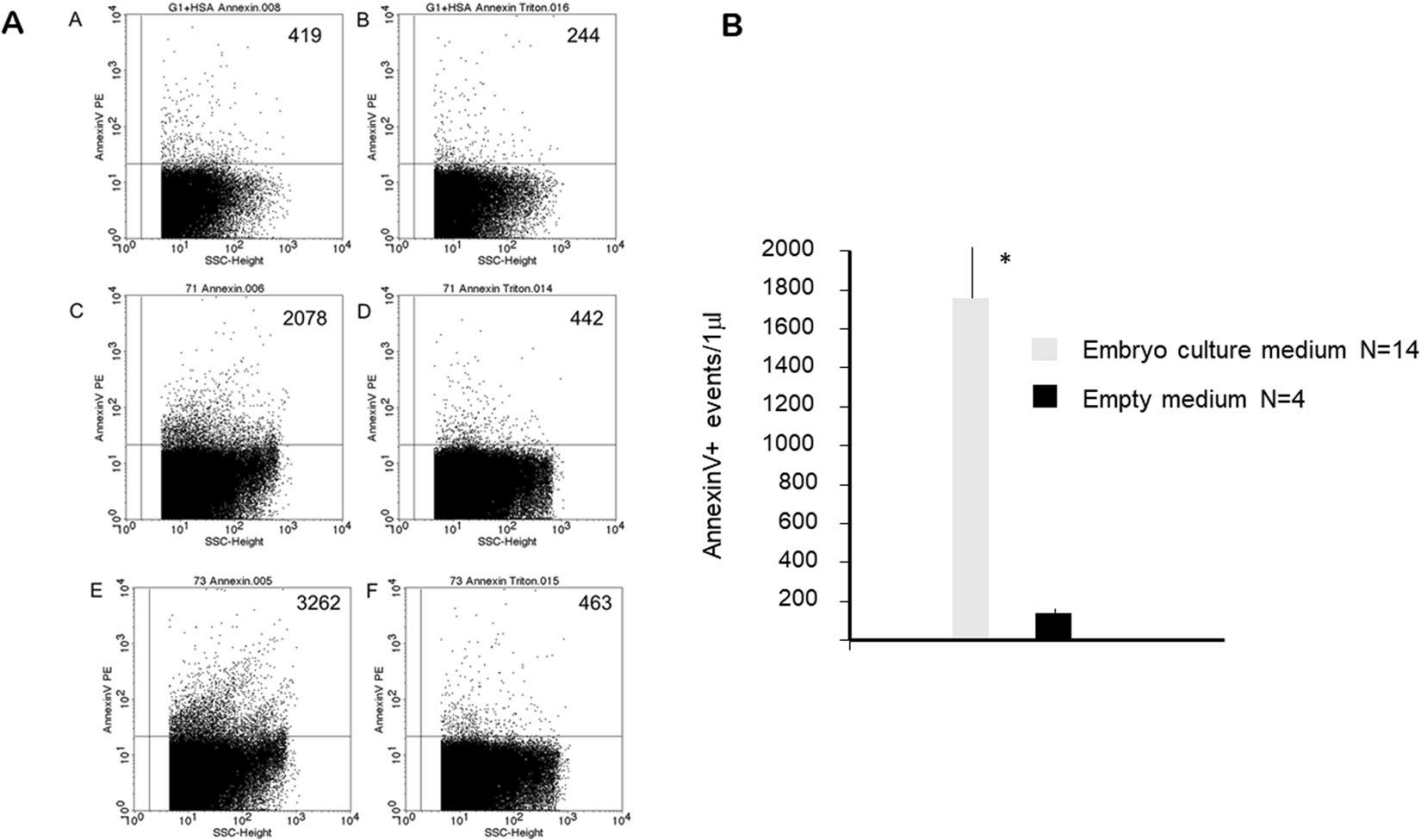
Extracellular vesicles in embryo culture media. EVs in mouse embryo culture media were identified by their Annexin V biding capacity, using flow cytometry. Annexin V containing empty medium was used for measuring the non-specific fluorescence background. The presence of EVs was confirmed by detergent lysis. **Panel A**; Annexin V positive EVs in two representative embryo culture media and an empty medium. (**A**) Annexin V positive EVs in empty medium. (**C**,**E**) Annexin V positive EVs embryo culture media. (**D**,**F**) Annexin V positive EVs disappear after TritonX treatment. (**B**)The background in empty medium is not sensitive to Triton X dependent lysis. Numbers in the upper right quadrants in panel A show the number of Annexin V+ events detected in a single measurement. **Panel B**; The number of AnnexinV labelled Evs/μl in 14 individual embryo culture media and 4 empty media. The bars represent the mean+/− SEM of 14 and 4 experiments respectively *p < 0.001.

Embryo-derived EVs in mouse embryo culture media were identified by their Annexin V biding capacity, using flow cytometry. Annexin V containing culture medium (without embryo) was used for measuring the non-specific fluorescence background (Fig. 1A in panel A). The number of Annexin V positive events was higher in embryo culture media (Fig. 1C,E in panel A), than in empty media (Fig. 1A in panel A). Annexin V positive EVs disappeared upon treatment of the samples with Triton-X (Fig. 1D,F in panel A), while the non-specific fluorescence background in empty medium was not sensitive to Triton-X dependent lysis (Fig. 1B in panel A). In further experiments the events sensitive to 0.1% Triton-X 100 were considered as EVs.

Analysis of 14 embryo culture media and 4 empty media revealed a significant difference (p < 0.001) in the number of Annexin V+ and TritonX-dependent lysis sensitive EVs between the two groups (Fig. 1 Panel B).

These data confirm, that that the cultured embryo produces detectable numbers of EVs, and Annexin V labelling is a reliable marker of embryo-derived EVs.

**Figure 2:**
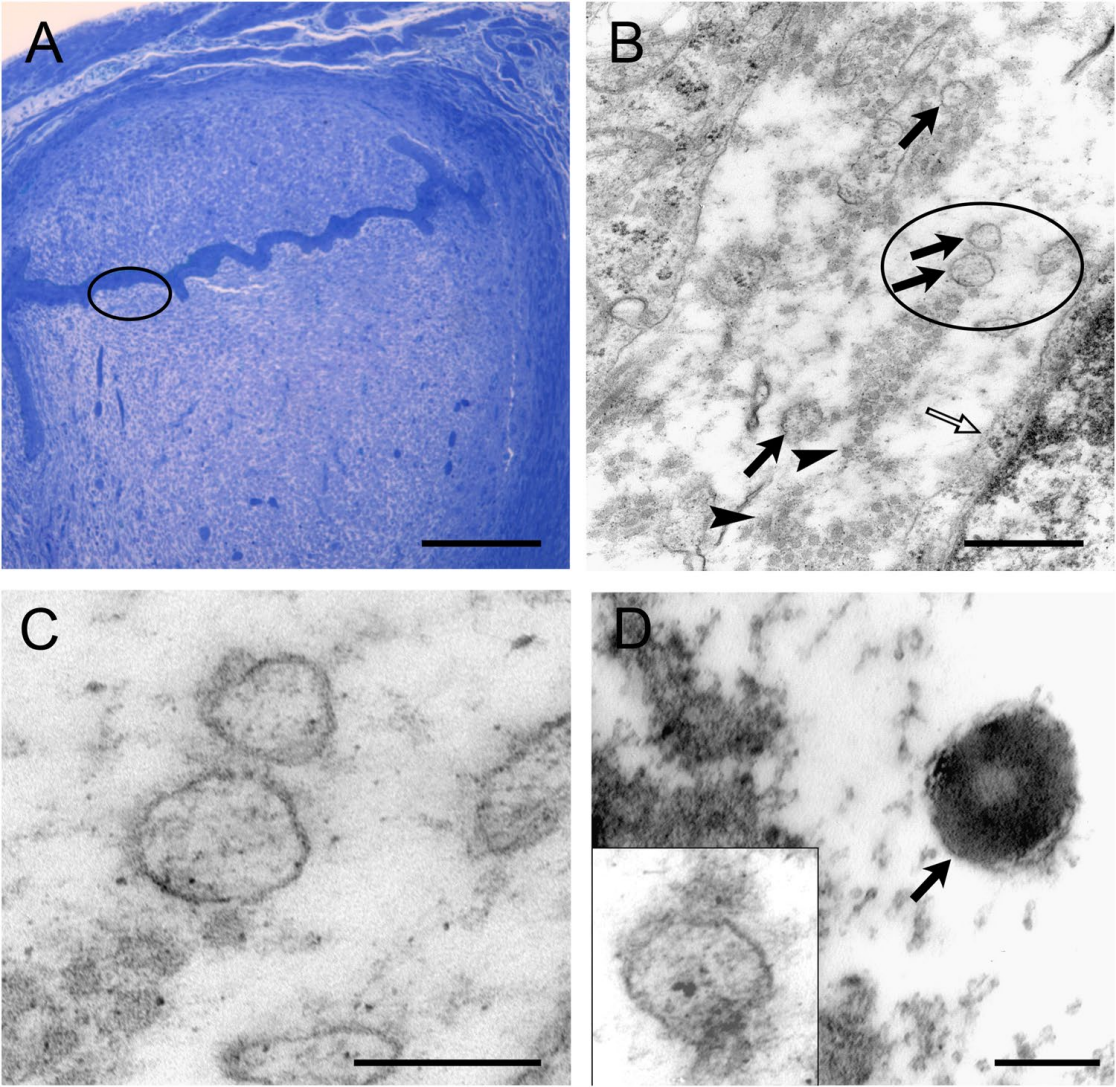
EVs at the feto-maternal interface and around *in vitro* cultured embryos. (**A**) Light microscopic photomicrograph showing implantation in a semi-thin section. Encircled area in A is shown with higher magnification in B. Scale bar = 200 μm. **(B)** Low magnification electron microscopic photomicrograph revealing EVs (arrows) at the embryo maternal interface. Open arrow point to the membrane of a maternal cell, while arrowheads indicate collagen fibres in the extracellular space. Encircled area in B is shown with higher magnification in C. Scale bar = 500 nm  (**C**) High magnification of EVs at the embryo maternal interface. Scale bar = 200 nm. (**D**) *In vitro* cultured morula stage mouse embryos were immune-stained with rabbit anti- PIBF primary antibody, then embedded in agar and prepared for electron microscopy. The EV (indicated with an arrow) produced by the embryo is PIBF-immuno-reactive. Insert shows EV reacted with the secondary antibody only, as a negative control. Scale bar = 100 nm.

EVs with a diameter of 100 to 200 nm were also demonstrated at the foeto-maternal interface by transmission electron microscopy in day 5 murine pregnancy (Fig. 2A–C).

PIBF expression in embryo-derived EVs was demonstrated with immuno-electron microscopy (Fig. 2D).

Our earlier data show that mouse embryos express PIBF at all stages of development (supplementary Fig. 2).

Previous flow cytometric analysis in our laboratory confirmed the presence of PIBF+ EVs in embryo culture medium. Direct labelling of unfixed samples, detects the surface bound PIBF molecules, while a direct labelling of paraformaldehyde fixed samples detects the intra-vesicular PIBF.

Twenty mouse embryo culture media were analysed by multicolour flow cytometry. After fixation of these samples by 4% paraformaldehyde solution, the vesicles were reacted with FITC-labelled PIBF antibody and propidium iodide (PI) at the same time. The double labelling revealed the presence of 1) PIBF+/PI− vesicles; 2) PIBF+/PI+ and 3) PIBF−/PI+ vesicles, suggesting that not all vesicles contain PIBF (Supplementary Fig. 3).

### Mouse embryo-derived extracellular vesicles bind to mouse spleen cells via phosphatidylserine

Binding of EVs to mouse spleen cells was confirmed by flow cytometry.

In addition to the fluorescence signals, FS (forward scatter) and SS (side scatter) parameters were also detected. Based on their size (FS) and granularity (SS) cells were classified as lymphocytes and non-lymphoid cells and gated accordingly (Supplementary Fig. 4) Fluorescence of PKH-labeled EV samples as well as of mock-stained control samples were assessed within the “non-lymphoid” and within the “lymphocyte” gates.

**Figure 3:**
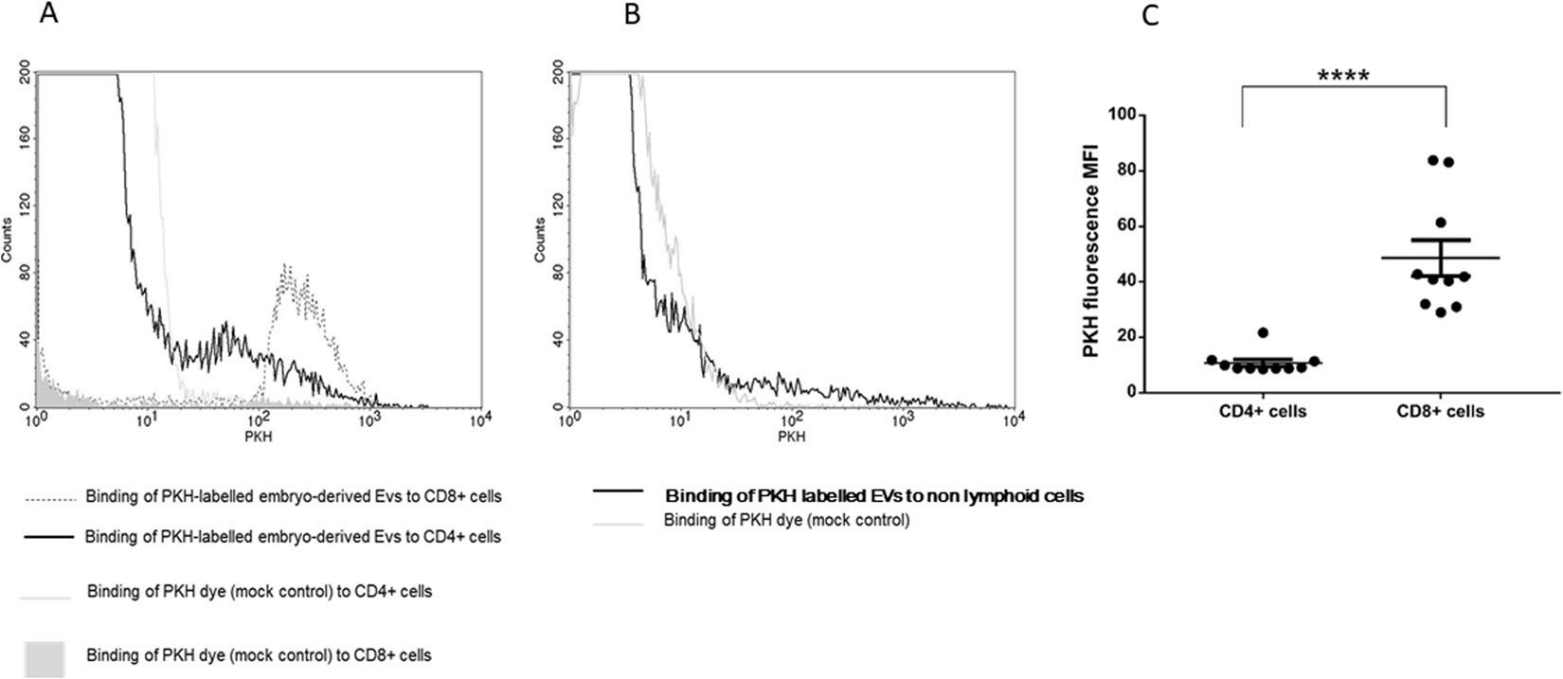
Binding of embryo-derived EVs to mouse peripheral lymphocytes. For comparing EV binding to different splenocytes including CD4+, CD8+, or „non-lymphoid” cells, PKH fluorescence intensity parameter (geometric mean channel values/MFI values) was used and overlay histograms were displayed. (**A**) Mock control inside CD8+ population is represented by a grey-filled histogram. Mock control inside CD4+ population is represented by the light grey line. EV binding to CD8+ cells is represented by unfilled grey dashed line. EV binding to CD4+ cells is represented by black line. (**B**) Mock control inside „non-lymphoid” population is represented by a grey histogram and EV binding is demonstrated by a dark line. (**C**) Significantly higher PKH fluorescence was detected within the CD8+ gate than in the CD4+ gate (p < 0.0001). The experiments were repeated 6 times in duplicates. Though it gave similar results, one of the experiments was excluded from this graph, because of the use of a different PKH dye. mean+/−SEM are shown.

Mouse embryo-derived EVs were found to adhere to the surface of both CD4+ and CD8+ T lymphocytes (Fig. 3A), but not to non-lymphoid cells (Fig. 3B).

For comparing the EV binding of CD4+ or CD8+ cells, we used the PKH fluorescence intensity parameter (geometric mean channel values/MFI values) which reflects on the number of bound EVs per cell. The mock control did not get any fluorescence signal at PKH wavelength. We defined CD4+ and CD8+ cells gates and compared the PKH fluorescence within the CD4+ and CD8+ lymphocyte gates. Significantly higher (p < 0.001) PKH fluorescence was detected in the CD8 + cells gate (MFI = 48.58 ± 6.19) than in the CD4+ cells gate (MFI = 10.81 ± 1.19), showing that CD8+ cells bound more embryo-derived EVs than CD4 + cells (Fig. 3C).

We hypothesized that EVs bind to the lymphocytes via an interaction between the membrane PS and the PSR on the surface of the cells.

**Figure 4:**
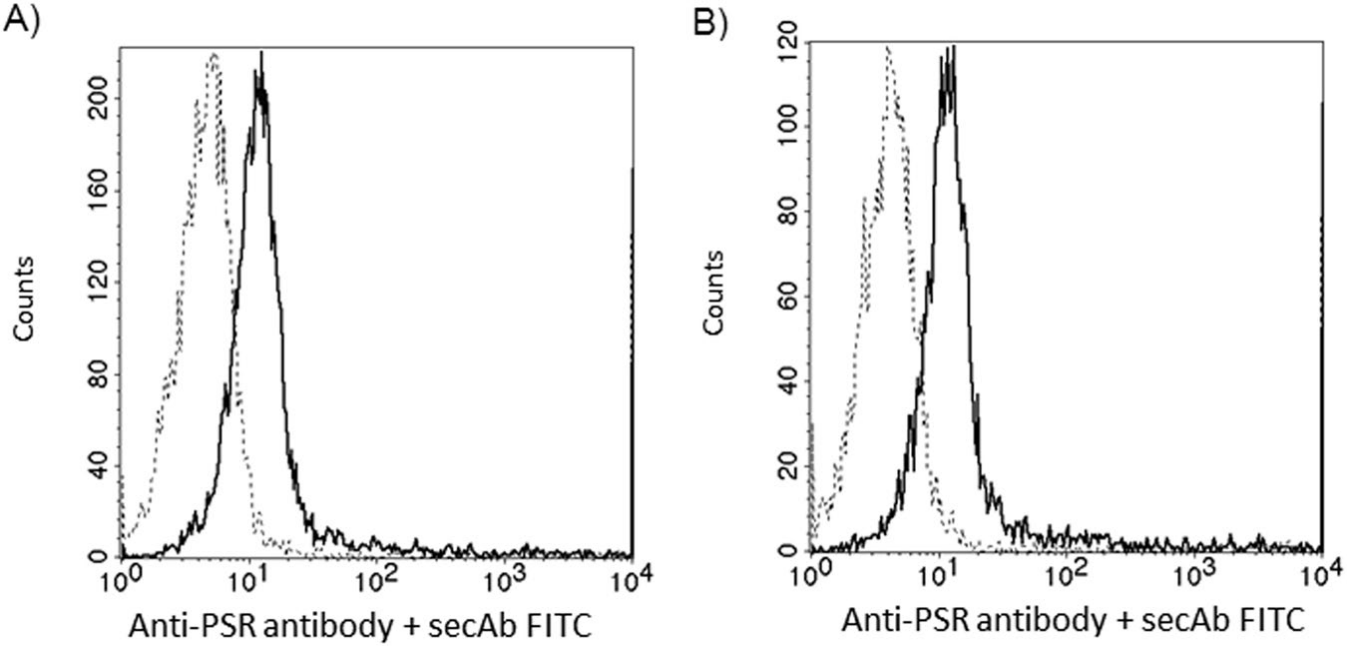
Phosphatidylserine receptor expression on CD4+ and CD8+ cells. Flow cytometry analysis of PSR expression on the cell surface of CD4+ (**A**) and CD8+ (**B**) T cells. Histograms show staining with unconjugated anti-PSR antibody followed by FITC-conjugated secondary goat anti-rabbit IgG (solid lines). Control cells were stained with FITC-conjugated secondary antibody alone (dashed lines).

Reacting CD4+ and CD8+ cells with anti-PSR antibody revealed an equal number of PSR/cell in the two subsets (Fig. 4)

**Figure 5:**
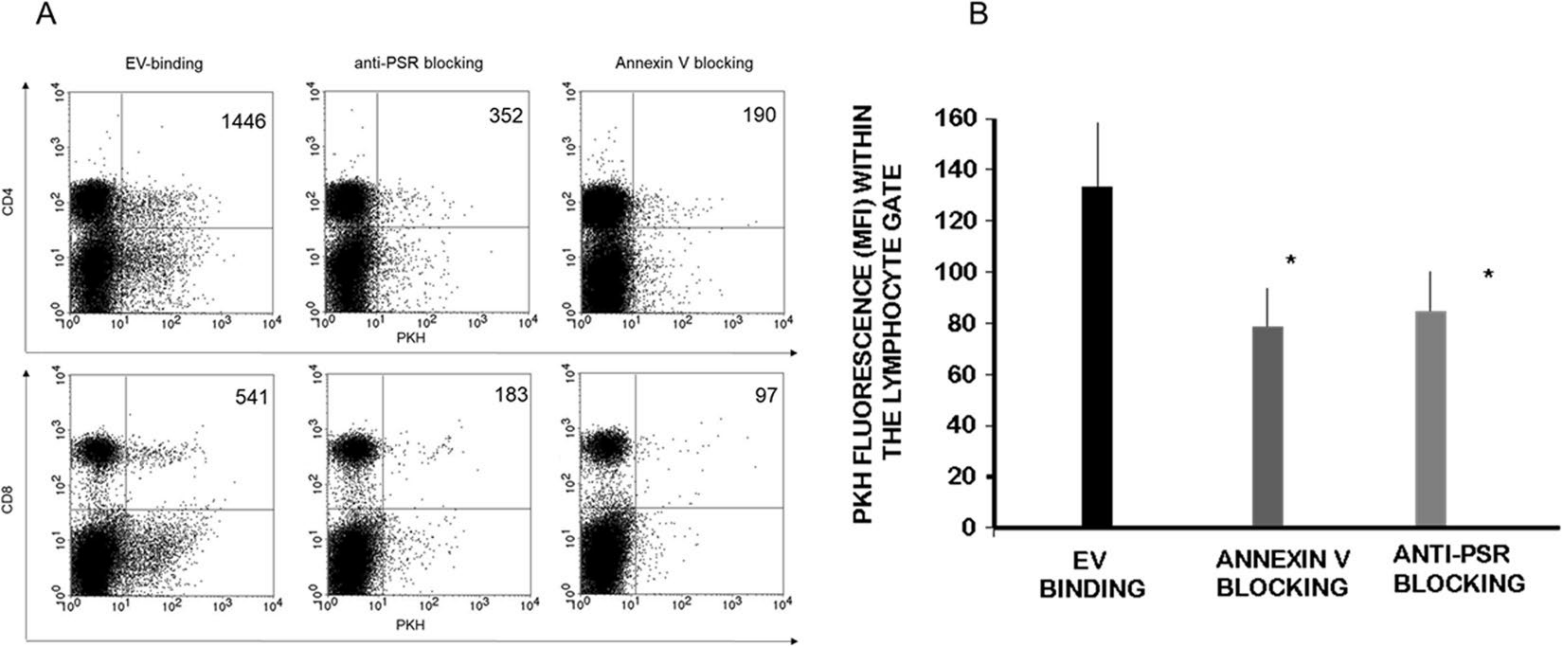
Blocking the exofacial phosphatidylserine binding sites of embryo-derived EVs inhibits the binding of EVs to the lymphocytes. Prior to incubating embryo-derived EVs with anti-CD4 and anti-CD8 labelled splenocytes, we masked phosphatidylserine on the membranes of EVs by AnnexinV, or the phosphatidylserine receptors on the lymphocytes and checked the number of EVs binding CD4+ and CD8+ cells. **Panel A**: The upper right quadrants of representative dot plots show the PKH+/CD4+ or PKH+ /CD8+ double positive cells. These populations represent the number of lymphocytes which bound PKH-labelled EVs on their surface. Both anti-PSR antibody pretreatment of lymphocytes, and blocking of the exofacial phosphatidylserine on embryo-derived EVs by Annexin V partially inhibited the binding of EVs. **Panel B**: For comparing EV binding of lymphocytes with or without blocking, we used the PKH fluorescence intensity parameter (geometric mean channel values/MFI values) which reflects on the number of EVs per cell. PKH fluorescence was measured in the lymphocyte gate. Significantly lower (p = 0.05) PKH fluorescence was detected both after anti-PSR antibody pretreatment and Annexin V blocking. The bars represent the mean+/− SEM of 4 experiments.

Therefore, we investigated the role of phosphatidylserine-phosphatidylserine receptor interaction in binding of embryo-derived EVs to T cells. Prior to incubating embryo-derived EVs with immune-phenotyped splenocytes, we masked the exofacial phosphatidylserine binding sites of embryo-derived EVs by AnnexinV, or the phosphatidylserine receptors on the lymphocytes by phosphatidylserine receptor specific monoclonal antibodies and checked the number of EV binding CD4+ and CD8+ cells. The binding of PKH-labelled embryo-derived EVs was partially inhibited in both cases (Fig. 5A). These experiments were repeated 4 times, and revealed a significant (p = 0.05) reduction in the number of EVs bound/cell following anti PSR or AnnexinV blocking (Fig. 5B). Therefore we concluded that PSR may be one of the target binding sites of embryo-derived EVs.

### PIBF containing EVs alter IL-10 expression of mouse peripheral lymphocytes

Anti-CD4 or anti-CD8 labelled mouse spleen cells were incubated with embryo derived EVs, or with EVs that had been treated with anti-PIBF antibody for 30 mins at 37 °C. Than the cells were labelled with anti- IL-10 antibody, and the number of IL-10 positive cells among the subpopulations was determined by flow cytometry.

**Figure 6:**
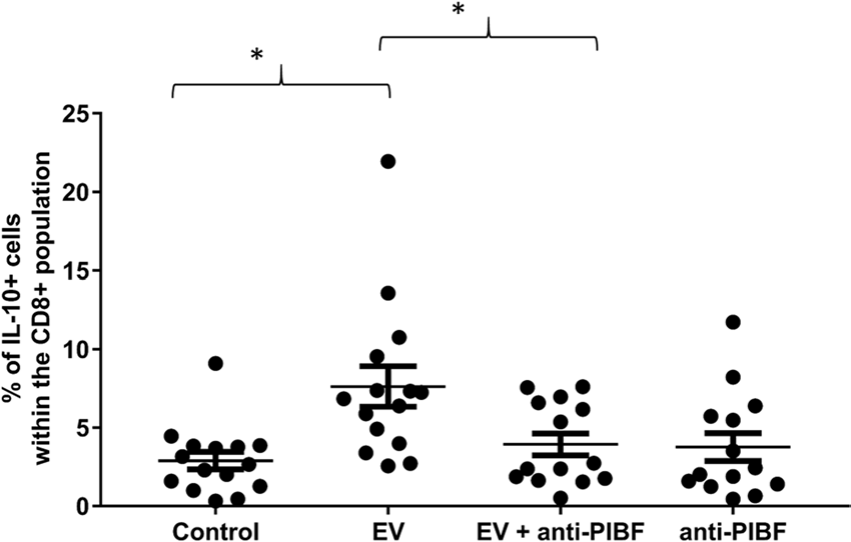
IL-10 expression of mouse peripheral CD8+ lymphocytes incubated with mouse embryo-derived EVs, or with anti-PIBF treated mouse embryo-derived EVs. Anti-CD8 labelled mouse spleen cells were incubated with embryo derived EVs, or with EVs that had been pre-treated with anti-PIBF antibody for 30 mins. Anti-rabbit IgG was used as an isotype control. Then the cells were labelled with anti- IL-10 antibody, and the number of IL-10 positive cells among the subpopulations was determined by flow cytometry. The bars represent the mean+/− SEM of 15 measurements. *p < 0.001.

The percentage of IL-10+ CD8+ cells increased in the presence of EVS, and this was counteracted by pre-treatment of EVs with an anti-PIBF antibody (Fig. 6). The presence of EVs did not alter IL-10 production in either CD4+ cells or of B cells (data not shown).

## Discussion

Here we show that mouse embryos produce extracellular vesicles both in culture and after implantation. EVs can be produced by virtually all cell types, however it has been debated, whether a single embryo would be able to produce a detectable amount of EVs. The more so, because the culture medium contains serum or albumin, both of which could also be a source of EVs. Indeed, Tannetta *et al*.^21^, points out the difficulty of measuring EVs in embryo culture medium.

For embryo culture we used KSOM medium supplemented 0.4% bovine serum albumin. When EV content of spent and empty media were compared (Fig. 1), though there was definitely a background in the empty medium, this was not sensitive to TritonX-dependent lysis, which excludes the presence of EVs in the empty medium, thus the observations are very likely due do the presence of embryo-derived EVs.

This is in line with our earlier data, showing that nucleic acid containing EVs are present in day 5 human embryo culture media, and that the number of these EVs is related to embryo competence^5^. Recently, other groups have also reported on the presence of EVs in embryo culture medium. Giacomini *et al*.^22^, characterized HLA-G containing EVs isolated from conditioned media from *in vitro* cultured human embryos. EVs were demonstrated in the culture medium of bovine blastocyst and the characteristics of these EVs varied depending on embryo competence^23^. Qi *et al*.^24^, showed that the negative effects of culture media replacement during embryo culture are due to the loss of embryo derived EVs, and can be corrected by exosome supplementation.

We have shown that EVs bind to the lymphocytes and the binding is inhibited both by anti-phosphatidylserine receptor antibodies and by Annexin V. Interestingly, we found no significant EV binding to non-lymphoid cells. This might be due to a lower phosphatidylserine receptor expression on these cells. However, the non-lymphoid population is a mixture of different cell types, and the phosphatidylserine receptor expression of each has not been tested.

Though CD4+ and CD8+ cells express similar numbers of phosphatidylserine receptor, CD8+ cells bound higher amounts of EVs than CD4+ lymphocytes, suggesting, that in addition to the PS-PSR binding, other, yet unidentified mechanisms might also be involved in binding of EVs to CD8+ cells.

EVs contain different kinds of molecules, including lipids, nucleic acids, peptides and proteins, and as such, they might act as vehicles transporting information from one cell to the other. EVs from both immune and non-immune cells might have important roles in immune regulation.

With immuno- electron microscopy we identified PIBF in embryo-derived EVs. PIBF mediates many of the immunological actions of progesterone during pregnancy. PIBF alters the arachidonic acid metabolism^25^, inhibits NK activity^26,27^ and results in Th2-dominant cytokine production by maternal lymphocytes^28^. Furthermore, owing to its immunological effects PIBF contributes to the maintenance of pregnancy. Higher rates of foetal loss were observed in mice treated with anti-PIBF compared to untreated controls^26^, and recent data show that, PIBF+ decidual B cells are needed for the maintenance of late pregnancy^29^.

We hypothesized that the intercellular communication between the embryo and the maternal immune system in the peri-implantation period might be established via EVs.

There is evidence for such a mechanism in more advanced pregnancy, as placental exosomes isolated from peripheral blood of pregnant women have been shown to suppress T-cell signalling^30^. In the present study, murine embryo-derived EVs, increased the number of IL-10+ cells among peripheral CD8+ cells, but not in the CD4+ population. IL-10 producing CD8+ T cells might have a special function. Earlier studies revealed that IL-10 producing CD8+ T cells control influenza virus induced inflammation in the lung^31^, and that virus-specific IL-10-producing CD8 T cells prevent liver damage during chronic hepatitis C virus infection^32^.

Pre-treatment of EVs with an anti-PIBF antibody abrogates the above effect of the EVs. These data suggest, that PIBF transported by the EVs from the embryo to maternal lymphocytes might induce increased IL-10 production by the latter, this way contributing to the Th2 dominant immune responses described during pregnancy. The finding is in line with our earlier data^33^, showing increased IL-10 production of murine spleen cells in the presence of PIBF.

We show for the first time, that murine embryo derived EVs bind to peripheral lymphocytes –at least in part - via phosphatidylserine, and influence IL-10 production of CD8 + peripheral lymphocytes. The latter effect can at least partly be attributed to PIBF content of the EVs.

These data suggest, that PIBF transported by the EVs from the embryo to maternal lymphocytes might induce increased IL-10 production by the latter, this way contributing to the Th2 dominant immune responses described during pregnancy.

The above findings contribute to our understanding of the embryo-maternal communication in the peri-implantation period.

## Electronic supplementary material

supplementary information
