# Supplementary material for: PIBF+ extracellular vesicles from mouse embryos affect IL-10 production by CD8+ cells
Source: Sci Rep. 2018 Mar 16;8:4662. doi: 10.1038/s41598-018-23112-z (PMC5856852; doi:10.1038/s41598-018-23112-z)
Supplement: Supplementary file 1 — supplementary information [file 41598_2018_23112_MOESM1_ESM.pdf]

PIBF+ extracellular vesicles from mouse embryos affect IL-10 production by CD8+ cells

Eva Pallinger[1], Zoltan Bognar[2,3,5], Agnes Bogdan[2,4,5], Timea Csabai [2,4,5], Hajnalka Abraham[2], Julia Szekeres-Bartho* [2,3,4,5]

1 Department of Genetics, Cell- and Immunobiology, Semmelweis University, Budapest

2 Department of Medical Biology, Medical School, Pecs University,

3 MTA - PTE Human Reproduction Research Group, Hungarian Academy of Science at Pecs University

4 János Szentágothai Research Centre, Pecs University, Pecs, Hungary.

5 Endocrine Studies, Centre of Excellence, Pecs University

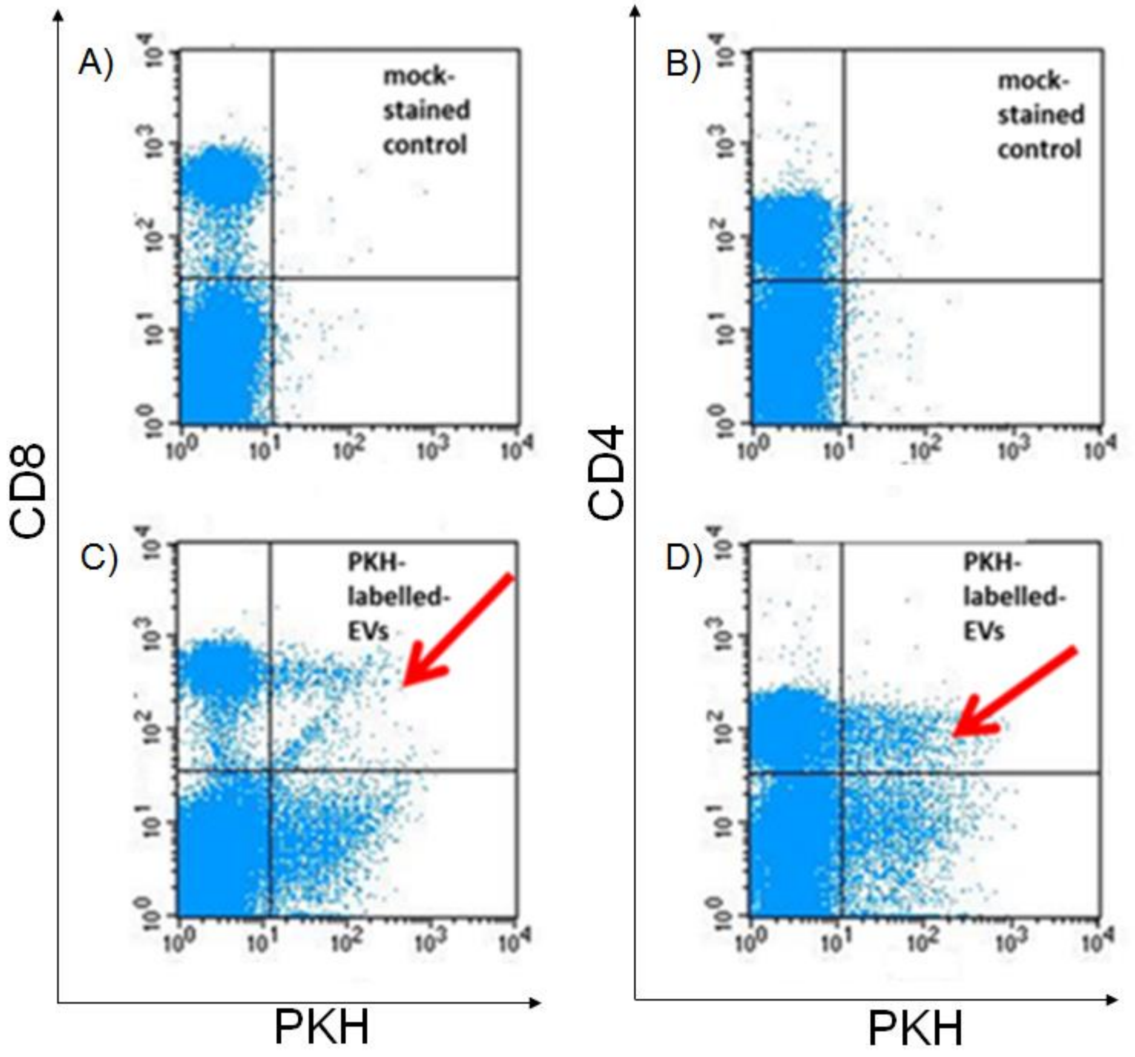

Supplementary Fig.1

Binding of PKH-labelled EVs to splenocytes:

To detect embryo-derived EVs binding to splenocytes, same volumes of PKH labelled EVs (1 x 10^6) or diluted PKH dye solution without vesicles (mock-stained control) were added to splenocytes

On the basis of their size (FS) and granularity (SS) lymphocytes and "non-lymphoid" cells were distinguished and gated. CD4+ and CD8+ T cells were identified inside the lymphocyte gate.

PKH+/CD4+ or PKH+/CD8+ double positive cells were analyzed and classified as EV-bound T lymphocyte subsets. Fluorescence of mock-stained control samples was always detected and compared to the fluorescence of PKH-labeled EV samples.

Representative figure shows that PKH fluorescence cannot be detected in the case of mock-stained control samples (PKH fluorescent dye without EVs).

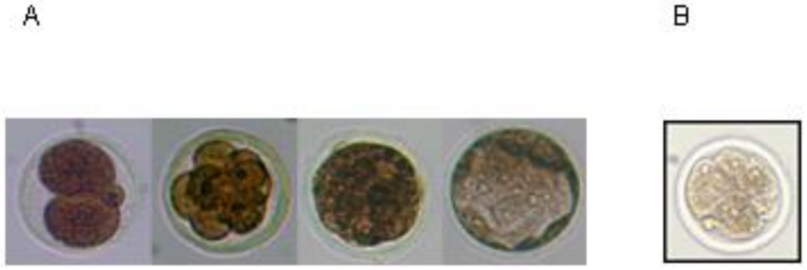

Supplementary Fig. 2

PIBF expression in murine embryos

In vitro cultured mouse embryos at different stages of development were reacted with anti-PIBF antibody and anti-rabbit HRPO (A), or anti-rabbit HRPO only (B)

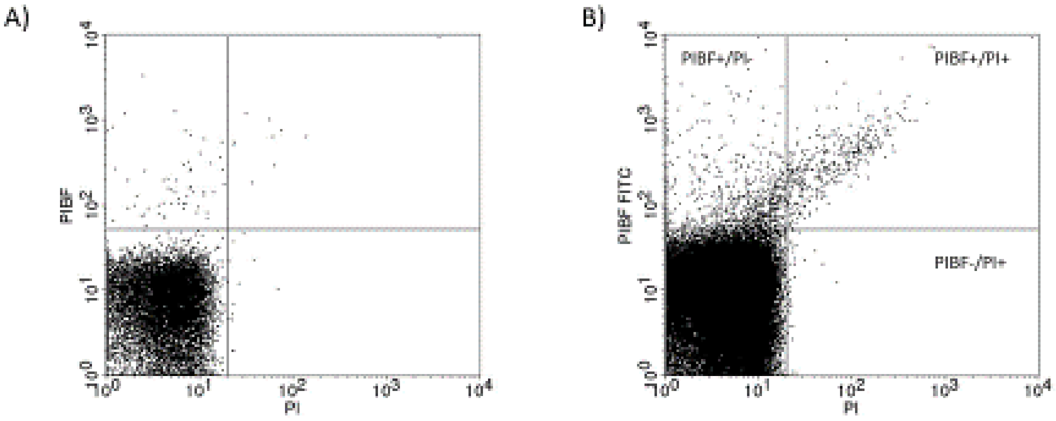

Supplementary Fig. 3

PIBF – propidium iodide double staining of embryo-derived EVs

A) Background fluorescence of empty IVF medium mixed with PI and FITC-anti-PIBF antibody.

B) Representative dot plot shows the propidium iodide (PI) and FITC-conjugated anti-PIBF antibody double staining of fixed EVs isolated from mouse IVF media.

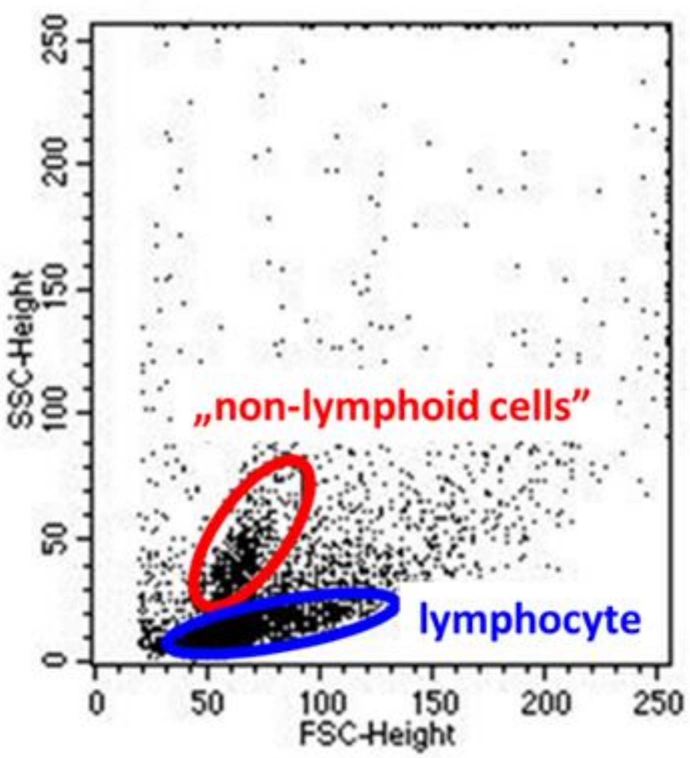

Supplementary Fig. 4

Size distribution of separated mouse spleen cells

In addition to the fluorescence signals, FS (forward scatter) and SS (side scatter) parameters were also detected. On the basis of their size (FS) and granularity (SS) spleen cells were gated and classified as lymphocytes and non-lymphoid cells
